# N-myc is a key switch regulating the proliferation cycle of postnatal cerebellar granule cell progenitors

**DOI:** 10.1038/srep12740

**Published:** 2015-08-04

**Authors:** Ming Ma, Wenting Wu, Qing Li, Jinya Li, Zhejin Sheng, Jiahao Shi, Mengjie Zhang, Hua Yang, Zhugang Wang, Ruilin Sun, Jian Fei

**Affiliations:** 1 School of Life Science and Technology, Tongji University. Shanghai 200092, China; 2 Shanghai Research Center for Model Organisms, Shanghai 201203, China

## Abstract

N-myc plays an important role in early cerebellar development; however, the role of N-myc in postnatal cerebellar development is still unknown. In this study, inducible and reversible N-myc mouse models (Nmyc^TRE/TRE^:tTS and Nmyc^EGFP/TRE^:tTS) are used to regulate and track the expression of endogenous N-myc *in vivo*. Loss of N-myc at the neonatal stage results in reduced proliferation of granule cell precursors (GCPs) and reduced cerebellar volume/mass. Restoration of N-myc expression no later than postnatal day 4 can rescue the cerebellar developmental defect caused by the absence of N-myc after birth. During cerebellar postnatal development, N-myc acts as a key switch, regulating the proliferation cycle of postnatal granule cell progenitors. Loss of N-myc significantly impairs the Sonic hedgehog signalling pathway, and disrupts the expression of cell cycle effectors with a significant reduction of Ccnd2. More importantly, N-myc negatively regulates the expression of microRNA-9 during postnatal cerebellar development. Our findings demonstrate that over-expression of miR-9 can inhibit the proliferation of GCPs. The regulation of these factors by N-myc is at least partly responsible for the switch role of N-myc in the proliferation cycle of GCPs.

The cerebellum is a well-defined anatomical structure of the brain, which controls coordination of movement, balance, equilibrium, and muscle tone by integrating signals from the spinal cord, the brain stem and the cerebral cortex with sensory input from the muscles and other areas^1^. The cerebellar granule cells are the most abundant neurons, not only within the cerebellum, but also within the entire mammalian central nervous system^2^. The development of the granule cells spans embryonic and postnatal development. In the embryonic stage, the granule cells originate from the rhombic lip^1^. In the rhombic lip, granule cell precursors (GCPs) proliferate and migrate from the rhombic lip to form the external granule layer (EGL)^1^. However, the bulk of proliferation of GCPs occurs postnatally^1^. Between the days 2 and 4 postpartum (P2–P4), a number of signalling pathways promote GCP proliferation^3^, which reaches a peak between postnatal days P4 and P8 and is completed by P15 in the mouse^1^,^2^. Between birth and the end of the second postnatal week, the GCPs exit the cell cycle, move into the inner regions of the EGL and migrate to a position beneath the Purkinje cells, where they form the inner granule layer^2^. In the first 2 weeks postpartum, the GCPs complete the bulk of their proliferation, differentiation and migration, becoming the most abundant neurons in the central nervous system. However, the molecular mechanisms that regulate the growth and differentiation of granule cells are not fully understood.

In previous studies, Sonic hedgehog (Shh) signalling has been shown to be a primary driver of the expansion of the GCP precursor pool^2^,^3^. N-myc, a transcription factor belonging to the MYC oncogene family, has been reported to be up-regulated by Shh, and to promote the proliferation of GCPs *in vitro*^4^. Loss of N-myc in neuronal progenitor cells by use of a nestin-Cre transgene severely disrupts cerebellar development, resulting in a significantly smaller and disorganised cerebellum, and particularly a significant reduction in granule cell numbers^5^. These results indicate that N-myc may play an important role in cerebellar development and granule growth. However, as loss of N-myc happens as early as embryonic day 9.5 (E9.5) in this conditional knockout mouse model^5^, the model is limited and cannot provide information about the role of N-myc in the growth and differentiation of the granule cells after birth.

In our previous work, an inducible and reversible endogenous N-myc gene expression mouse model was developed^6^, which provides an opportunity to explore the function of N-myc in a specific time window. Using this model, we have shown that loss of N-myc after birth also results in defective cerebellar development, a smaller cerebellum and a reduction of granule cell density^6^. Significantly, we demonstrated that the defects caused by loss of N-myc can be rescued by restoring N-myc expression^6^. These data indicate that N-myc also plays an important role in postnatal cerebellar development. However, the exact role of N-myc in postnatal cerebellar development and the underlying molecular mechanisms are still unclear.

In this study, by using our inducible and reversible N-myc mouse model, we demonstrate that the defects in cerebellar development resulting from N-myc loss are mainly owing to the reduction of granule cells. This defect in cerebellar development can be rescued if N-myc expression is restored no later than 4 days postpartum. We show that N-myc is highly expressed in the proliferating GCPs in the cerebellar external granular layer. N-myc acts as a key switch to regulate the turnover of the GCP proliferation cycle. Loss of N-myc changes the expression of factors in the Shh signalling pathway, and disrupts the normal expression of cell cycle factors with a significant reduction of Cyclin D2 (Ccnd2) protein levels. More importantly, we demonstrate that N-myc negatively regulates the expression of microRNA-9 during postnatal cerebellar development, and over-expression of miR-9 *in vivo* can inhibit the proliferation of GCPs. The regulation of these factors by N-myc is at least partly responsible for the switch role of N-myc in the proliferation cycle of GCPs.

## Results

### Silencing N-myc expression severely disrupts postnatal cerebellar development

Nmyc^TRE^ mice and tTS transgenic mice were developed as previously described^6^. In Nmyc^TRE^ mice, a tetracycline response element (TRE) sequence was inserted into the first intron of the endogenous N-myc gene. In tTS mice, an artificial transcriptional silencer, tetracycline-controlled transcriptional suppressor (tTS), was ubiquitously expressed, which can recognise the TRE site. In Nmyc^TRE/TRE^:tTS mice, N-myc expression was dependent on the presence or absence of doxycycline (DOX). In the absence of DOX, tTS binds to the TRE site and N-myc transcription is repressed. In the presence of DOX, tTS binds to DOX and N-myc shows normal expression.

**Figure 1:**
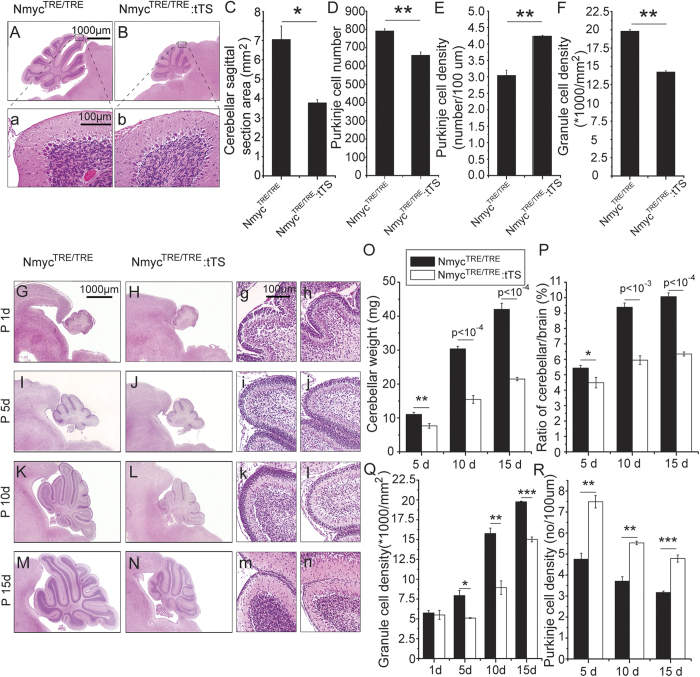
Silencing N-myc expression severely disrupts postnatal cerebellar development. (**A**, **a**) HE staining of cerebellum sagittal section from TRE mice. (**B**, **b**) HE staining of cerebellum sagittal section from tTS mice. (**C**) The difference in cerebellar sagittal section area size between tTS and TRE mice. (**D**) The difference in Purkinje cell number between tTS and TRE mice. (**E**) The difference in Purkinje cell density between tTS and TRE mice. (**F**) The difference in granule cell density between tTS and TRE mice. (**G**–**N**) The cerebellar development stages of tTS and TRE mice from postnatal 1 d to 15 d. (**O**–**R**) Analysis of cerebellar weight, cerebellum/brain ratio, granule cell density and the Purkinje cell density between tTS and TRE mice at postnatal 5 d, 10 d, 15 d (n = 5). ***p < 0.001; **p < 0.01; *p < 0.05.

Nmyc^TRE/TRE^:tTS mice (abbreviated as tTS mice) and their littermates (Nmyc^TRE/TRE^, termed TRE mice) were exposed to DOX from embryo day 0.5 (E0.5) to E6.5 by adding DOX in drinking water, resulting in N-myc expression. This procedure guaranteed the birth of living pups by activating N-myc expression during embryo development, and postnatal expression of N-myc was subsequently inhibited in tTS mice^6^. It was found that the cerebellum of tTS mice was smaller than that of TRE mice (Fig. 1A–b) at 6 weeks. This was consistent with a significantly reduced cerebellar area (~50%) and granule cell density (~35%) in tTS mice compared with TRE mice (Fig. 1C,F). Interestingly, the number of Purkinje cells was decreased (~20%) but the density was increased (~20%) in tTS mice compared with TRE mice (Fig. 1D,E), because of the smaller cerebellar volume of tTS mice.

To further explore the time point of the cerebellar defect, the cerebellum was harvested at different postnatal points from tTS and TRE mice. There was no significant morphological difference in the cerebellum between tTS and TRE mice (Fig. 1G–h) before P5 (Fig. 1I–j). The cerebellar weight, ratio of cerebellar weight/brain weight and granule cell density were gradually but significantly decreased in tTS mice from P5 to P15 compared with TRE mice (~2-fold, ~1.5-fold and ~1.9-fold, respectively, at day 10). Similarly, the Purkinje cell density was gradually and significantly higher in tTS mice than TRE mice at the above three time points (~1.4-fold at day 10) (Fig. 1R).

### Restoring N-myc expression no later than postnatal day 4 is critical for cerebellar development

**Figure 2:**
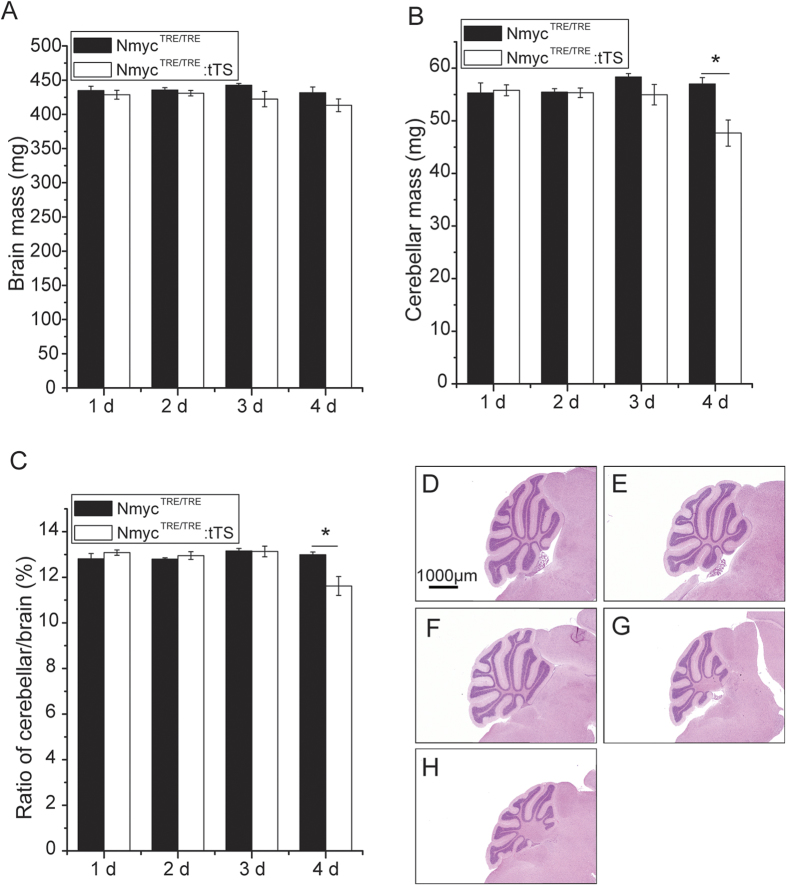
Time point for N-myc re-expression to rescue postnatal cerebellar development. The N-myc expression of tTS mice was re-activated by DOX treatment at 1 d, 2 d, 3 d, or 4 d postpartum. After 6 weeks they were sacrificed and the difference in cerebellar development was investigated and compared with TRE mice. (**A**) The brain mass of mice from the DOX-treated tTS group and TRE mice. (**B**) The cerebellar mass of mice from the DOX-treated tTS group and TRE mice. (**C**) The weight ratio of the cerebellum/brain of tTS and TRE mice. (**D**) The HE staining of the cerebellum from TRE mice. (**E**–**H**) The HE staining of the cerebellum from tTS mice separately treated with DOX at postnatal 1 d, 2 d, 3 d, and 4 d (n = 9 from each group). *p < 0.05.

To further confirm the functional time window for N-myc expression on postnatal cerebellar development, a rescue experiment was performed. tTS mice were exposed to DOX to activate N-myc from E0.5 to E6.5, and then were re-exposed to DOX to re-activate N-myc at either P1, P2, P3 or P4. There was no significant difference in brain weight between the tTS mice and TRE mice at the different exposed and re-exposed DOX points when the brains were harvested from 6-week-old mice (Fig. 2A). Moreover, there was no significant difference in cerebellar mass among the P1, P2, or P3 DOX re-treated mice and TRE mice. However, a significantly decreased cerebellar weight (~21%, *p* < 0.05) and weight ratio of cerebellum/brain (~15%, *p* < 0.05) were observed only on DOX re-exposure at P4, compared with TRE mice (Fig. 2B,C). This was confirmed by hematoxylin and eosin (HE) staining of cerebellum sections from the P4 group, which showed a smaller cerebellar structure than that of other groups (Fig. 2D–H). Considering that N-myc expression will have been restored after 1 day of exposure to DOX, the data suggest that re-expression of N-myc no later than postnatal day 4 is critical for cerebellar development. The cerebellar developmental deficiency is irreversible if N-myc is not restored before postnatal day 4.

### N-myc is a key switch in the proliferation of cerebellar granule neuron precursors

Nmyc^EGFP/+^ mice (abbreviated as EGFP mice) were developed as previously described^7^. When TRE was inserted, an enhanced green fluorescent protein (EGFP) cassette was fused in frame into exon 2 of N-myc, downstream of the first three amino acid coding sequence (ATGCCCAGC), to monitor transcription of N-myc.

**Figure 3:**
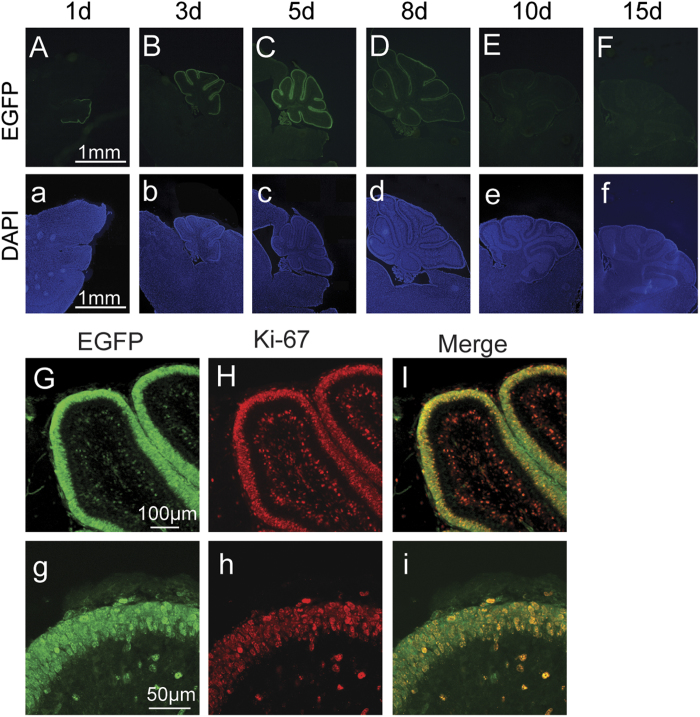
Expression pattern of N-myc in the postnatal cerebellum. (**A**–**F**) N-myc (EGFP) expression in the developing cerebellum in p1d (postnatal day 1), p3d, p5d, p8d, p10d, p15d. (**a**–**f**) 4′,6-Diamidino-2-phenylindole (DAPI) staining of the cerebellum. (**G**, **g**) N-myc (EGFP) expression in the external granular layer at p8d. (**H**, **h**) The Ki-67 immunofluorescence in the external granular layer at p8d. (**I**, **i**) Merged image of the EGFP and Ki-67 signals.

To investigate the expression pattern of N-myc in the postnatal cerebellum, cerebella from different postnatal days were harvested and observed. As shown in Fig. 3A–F, the EGFP signal was observed in the external granular layer, but not the inner granule layer. The EGFP signal increased gradually from P1 to P5 (Fig. 3A–C), and reached a plateau at P8 (Fig. 3D). Trace levels of EGFP were still detectable at P15 in the cerebellar granule neuron precursors of the external granular layer (Fig. 3F). Ki-67^+^ cells (a marker of proliferation) co-localised with the EGFP signal in the cerebellar granule cell precursors at P8 (Fig. 3G–i), indicating that N-myc is highly expressed in the proliferating GCPs.

Nmyc^EGFP/TRE^:tTS mice (abbreviated as EGFP:tTS) were obtained by crossing Nmyc^EGFP/+^ mice with Nmyc^TRE/+^:tTS mice. In the EGFP:tTS mouse, N-myc and EGFP expression is regulated by tTS, depending on the presence or absence of DOX. The presence or absence of N-myc expression can be reported by EGFP fluorescence.

**Figure 4:**
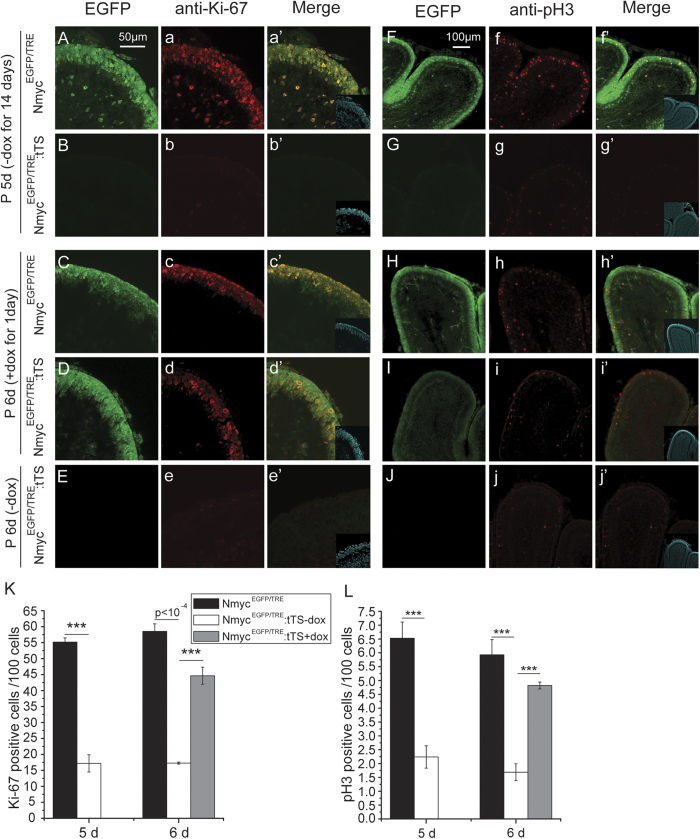
N-myc is a key switch in the proliferation of cerebellar granule neuron precursors. (**A**–**a**’) The expression of N-myc (EGFP) and Ki-67 in cerebellar granule neuron precursors of TRE:EGFP mice at p5d. (**B**–**b**’) N-myc (EGFP) and Ki-67 expression in few cerebellar granule neuron precursors of tTS:EGFP mice at p5d. (**F**–**f**’) The expression of N-myc (EGFP) and pH3 in cerebellar granule neuron precursors of TRE:EGFP mice at p5d. (**G**–**g**’) N-myc (EGFP) and pH3 expression in few cerebellar granule neuron precursors of tTS:EGFP mice at p5d. (**C**–**c**’, **H**–**h**’) The expression of N-myc (EGFP), Ki-67 and pH3 in cerebellar granule neuron precursors of TRE:EGFP mice at p6d. (**D**–**d**’, **I**–**i**’) The expression of N-myc (EGFP), Ki-67 and pH3 in cerebellar granule neuron precursors of DOX re-treated tTS:EGFP mice at p6d. (**E**–**e**’, **J**–**j**’) The expression of N-myc (EGFP), Ki-67 and pH3 in cerebellar granule neuron precursors of tTS:EGFP mice at p6d. (**K**) The quantification of Ki-67. (**L**) The quantification of pH3 (n = 5). ***p < 0.001. The insets are nuclear staining by DAPI.

To explore whether the proliferation of the cerebellar granule cell precursors depends on the expression of N-myc, EGFP:tTS mice were exposed to DOX to activate N-myc expression from E0.5 to E9.5, and then withdrawn on E9.5 to deactivate N-myc expression. N-myc expression in the external granular layer of EGFP:tTS mice was absent at P5 (Fig. 4B,G), which was consistent with decreased Ki-67^+^ and pH3^+^ cells (markers of proliferation) (~73% and ~65%, respectively) (Fig. 4B–b’,G–g’,K,L). Conversely, the Ki-67 and pH3 signals were highly co-localised with EGFP in the Nmyc^EGFP/TRE^ mice (EGFP:TRE mice) at P5.

To further determine if N-myc re-expression can restore the proliferation of cerebellar GCPs, EGFP:tTS mice were exposed to DOX from E0.5 to E9.5 and then DOX was removed from E9.5 until P5. Some of the EGFP:tTS mice were then re-exposed to DOX for 1 day at P5, and the other EGFP:tTS mice received no DOX as a control. At P6, in the EGFP:tTS mice that were re-exposed to DOX, EGFP expression was restored, and the percentage of Ki-67 and pH3 positive cells was 80% of that seen for Nmyc^EGFP/TRE^ mice (Fig. 4C–d’,H–i’,K,L). These data show that GCPs exit the proliferation cycle on loss of N-myc, and that restoration of N-myc expression promotes the re-entry of the GCPs into the proliferation cycle.

### Loss of N-myc promotes differentiation of cerebellar GCPs

**Figure 5:**
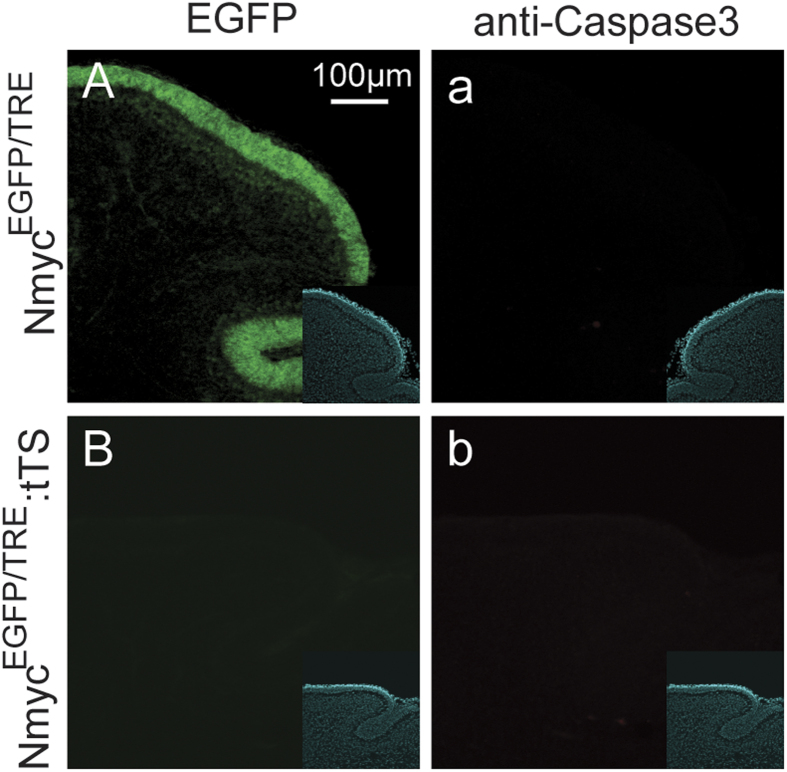
Loss of N-myc failed to induce the apoptosis of cerebellar granule neuron precursors. (**A**) The N-myc (EGFP) expression in the cerebellar granule neuron precursors of TRE:EGFP mice at p6d. (**a**) There was no caspase3 expression in the cerebellar granule neuron precursors of TRE:EGFP mice at p6d. (**B**) There was no N-myc (EGFP) expression in the cerebellar granule neuron precursors of tTS:EGFP mice at p6d. (**b**) There was no caspase3 expression in the cerebellar granule neuron precursors of tTS:EGFP mice at p6d. The insets are nuclear staining by DAPI.

**Figure 6:**
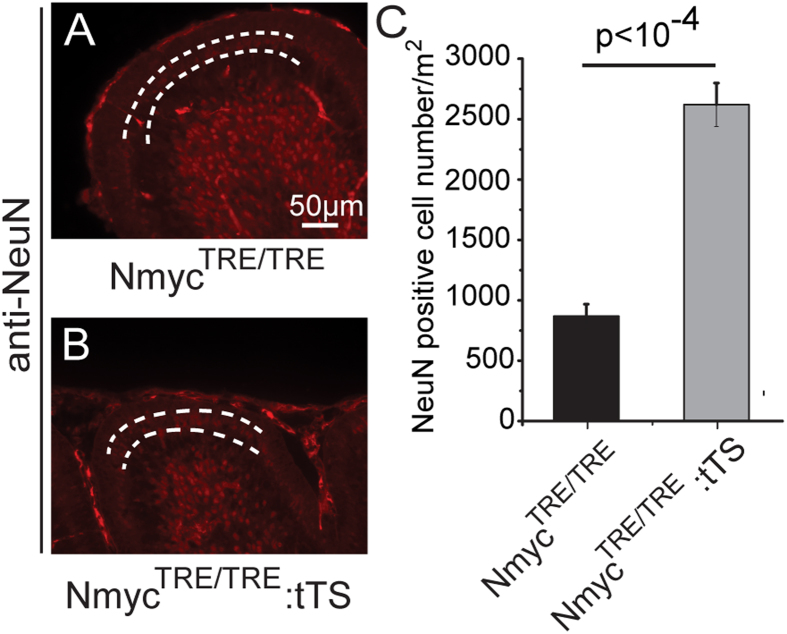
Inhibition of N-myc promoted the differentiation of cerebellar granule neuron precursors. (**A**) NeuN staining shows the differentiating GCPs population (between the white dotted lines) in the inner of EGL of TRE mice (**A**) and tTS mice (**B**). (**C**) The quantification of NeuN-positive cells in TRE mice and tTS mice (n = 5).

To further explore the fate of GCPs after loss of N-myc, apoptosis and differentiation assays were performed on the GCPs. EGFP:tTS mice were exposed to DOX to activate N-myc expression from E0.5 to E6.5, and then DOX was withdrawn to deactivate N-myc expression. N-myc expression was turned off after 2 weeks of DOX withdrawal. At day 6 postpartum, no increased apoptosis was observed in the external granular layer of EGFP:tTS mice (loss of N-myc after birth) compared with TRE:EGFP mice (normal expression of N-myc) (Fig. 5A–b). NeuN, a neuronal differentiation marker, was used to detect cell differentiation. NeuN^+^ cells were increased in the inner of the external granular layers (the cells between the white dotted lines), suggesting differentiation of cerebellar granule neuron precursors (Fig. 6A–C). These data indicate that loss of N-myc promotes the differentiation of cerebellar GCPs, but does not induce apoptosis of the GCPs.

### Loss of N-myc in GCPs disrupts the Shh signalling pathway and cyclin expression

**Figure 7:**
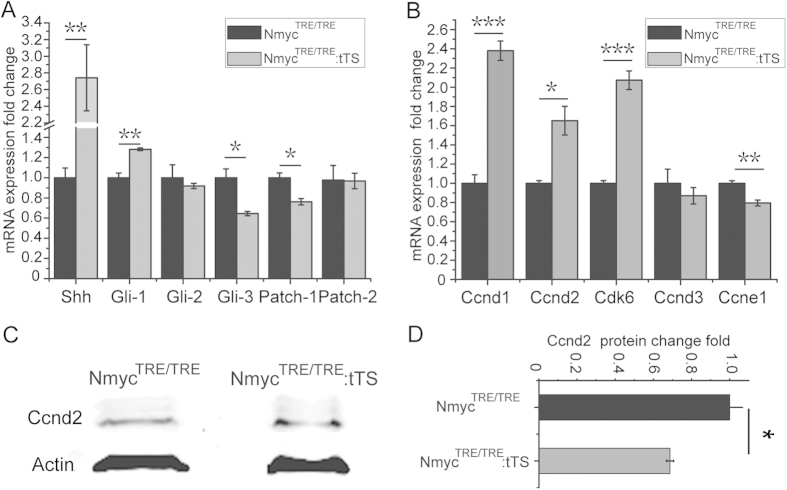
Loss of N-myc in GCPs disrupts the Shh signalling pathway and cyclin expression. (**A**) The expression level of Shh signalling pathway upstream members in tTS mice and TRE mice. (**B**) The expression level of cyclin family members in the GCPs of tTS mice and TRE mice. (**C**) Western blotting assay of Ccnd2 expression in the GCPs of tTS mice and TRE mice. (**D**) Western blotting quantification of Ccnd2 expression. The assay was independently repeated three times. *p < 0.05.

Next, the changes to upstream effectors of the Shh signalling pathway after loss of N-myc were assayed. tTS mice were exposed to DOX to activate N-myc expression from E0.5 to E6.5, and this was then withdrawn on E6.5 to deactivate N-myc expression. At P7.5, after loss of N-myc for 7 days, the Shh mRNA level in the cerebellum of tTS mice was significantly increased to 2.7-fold levels in TRE mice (Fig. 7A). In GCPs, Gli-1 mRNA expression levels increased by 30% in tTS mice, while Gli-3 and Patch-1 expression in GCPs was significantly reduced by 36% and 24%, respectively; no significant changes in Gli-2 and Patch-2 expression were observed in tTS mice (Fig. 7A).

To further explore the mechanism by which loss of N-myc inhibits the proliferation cycle of GCPs, the expression of cyclins related to the cell cycle was examined. At P7.5, the mRNA levels of CyclinD1 (*Ccnd1*), CyclinD2 (*Ccnd2*), CyclinD3 (*Ccnd3*) and CyclinE1 (*Ccne1*) in GCPs after loss of N-myc for 7 days were determined using quantitative reverse-transcription polymerase chain reaction (qRT-PCR). The expression of *Ccne1* was reduced by 30%, and no significant change of *Ccnd3* was seen in cerebellar granule neuron precursors from tTS mice compared with TRE mice (*p* < 0.01) (Fig. 7B). However, the expression levels of *Ccnd1* and *Ccnd2* were 2.4- and 1.7-fold higher, respectively, than the levels in TRE mice (*p* < 0.05) (Fig. 7B). The expression of cyclin-dependent kinase-6 (*Cdk6*), which can combine with Ccnd1 and Ccnd2^8^,^9^,^10^, was also up-regulated (2.1-fold) (Fig. 7B). The protein level of Ccnd2 in GCPs after loss of N-myc was determined by western blotting (Fig. 7C). In contrast to the RNA data, a significant 30% reduction of Ccnd2 protein expression was observed in tTS mice (Fig. 7D).

### N-myc negatively regulates the expression of miR-9 in GCPs

**Figure 8:**
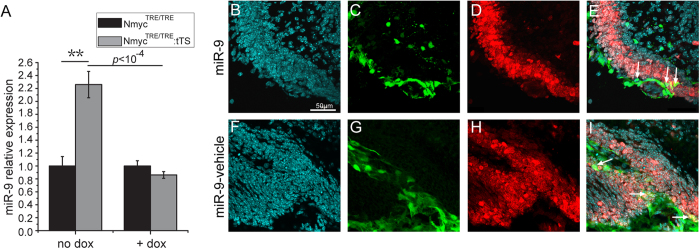
miR-9 negatively responds to N-myc expression, and the over-expression of microRNA-9 inhibits the proliferation of cerebellar granule neuron precursors *in vivo.* (**A**) miR-9 expression changes in the absence and presence of DOX. (**B**) DAPI staining of cerebellar granule neuron precursors. (**C**) Cerebellar granule neuron precursors infected with miR-9 over-expression lentivirus vector (EGFP-marked). (**D**) Ki-67 staining of cerebellar granule neuron precursors. (**E**) Merged image of (**B**–**D**). (**F**) DAPI staining of cerebellar granule neuron precursors. (**G**) Cerebellar granule neuron precursors infected with vehicle lentivirus vector (EGFP marked). (**H**) Ki-67 staining of cerebellar granule neuron precursors. (**I**) Merged image of (**F**–**H**). White arrows indicate the representative granule cell precursors infected with miR-9 or miR-9 vehicle over-expression lentivirus.

To investigate the mechanism by which N-myc regulates the proliferation of GCPs, microRNAs (miRNAs) involved in cell proliferation were also examined. tTS mice were exposed to DOX to activate N-myc expression from E0.5 to E6.5, and this was then withdrawn on E6.5 to deactivate N-myc expression. At P7.5, after loss of N-myc for 7 days, a total of 19 miRNAs were screened using qRT-PCR in cerebellar GCPs. miR-9 expression was up-regulated more than 2-fold in the GCPs of tTS mice (Fig. 8A), but was reduced to a normal level at P7.5 in the cerebellar granule neuron precursors of tTS mice treated with DOX since P5 (Fig. 8A). The miR-9 expression level also showed a significant difference between tTS mice with or without DOX treatment (Fig. 8A). These results suggest that N-myc negatively regulates the expression of miR-9 during postnatal cerebellar development.

### miR-9 inhibits proliferation of cerebellar GCPs *in vivo*

To study whether miR-9 can regulate the proliferation of cerebellar GCPs, miR-9 or a vehicle over-expression lentiviral vector labelled with EGFP were microinjected into the cerebellum of C57 mice at P4. Four days after microinjection, the proliferation statuses of the GCPs that were transfected with either miR-9 or the vehicle were examined. The miR-9 over-expression GCPs showed almost no Ki-67 positive signal (30 of 32, 93.75%, *p* < 0.05) (Fig. 8B–E), while GCPs transfected by the vehicle lentivirus generally showed Ki-67 positive signals (Fig. 8F–I). These results indicate that miR-9 can inhibit the proliferation of GCPs *in vivo*.

## Discussion

Conditional inactivation of N-myc during early embryonic cerebellar development leads to a severe defect in cerebellar organogenesis and a significant reduction of GCPs *in vivo*^5^. However, the bulk proliferative phase of the cerebellum in normal development occurs postnatally, which has raised the question of whether N-myc still plays a key role in cerebellum development postnatally. In this report, we demonstrate for the first time that loss of N-myc after birth precludes the proliferation of cerebellar GCPs, resulting in severe cerebellar developmental defects, with reduction of granule cells and a small cerebellar mass. Further rescue experiments show that restoration of N-myc expression can avoid cerebellar development defects, if this restoration occurs no later than 4 days postnatally. Postnatal day 4 was the starting point of the N-myc functional time window, which was also the starting point of the bulk proliferation^1^. In our previous study, we observed that the level of defect was unchanged whether or not N-myc was restored later than P11^6^. Combined with these earlier findings, we conclude that N-myc still plays an important role in postnatal cerebellar development, and its functional time window is P4–P11.

It has been reported that N-myc regulates cell cycle progression in cerebellar GCPs^4^. However, the exact role of N-myc in regulating cell cycle progression is unknown. In our study, postnatal loss of N-myc resulted in GCPs exiting the cell cycle. After restoration of N-myc expression, these GCPs re-entered the proliferation cycle. These findings indicate that N-myc plays a role as a switch, regulating the turnover of GCPs. GCPs that exited the cell cycle as a result of N-myc loss during postnatal days 1 to 5 retained the capacity to proliferate. However, after postnatal day 4, the restored expression of N-myc did not completely rescue the cerebellar developmental defect. It is unclear whether this is because the previous loss of N-myc impairs the capacity for proliferation on restoration, or whether there is a fixed end to the window in which GCPs can proliferate that cannot be extended.

The reduction of cerebellar granule cell density owing to postnatal loss of N-myc could be caused by increasing apoptosis or precocious differentiation of GCPs. No obvious apoptosis signal was detected in the P6 cerebellum (loss of N-myc since birth). An increased NeuN positive signal was observed in the inner layer of the EGL, suggesting a precocious differentiation of GCPs, but no signal was observed in the outer layer cells of the EGL. These findings suggest that N-myc expression inhibits neuronal differentiation of GCPs. Loss of N-myc could accelerate the tendency of GCPs to differentiate, but it is not the key regulator that decides the differential fate of the GCPs.

D-type cyclins and Cyclin-dependent kinase inhibitors have been identified as potential targets for Myc-mediated regulation^11^,^12^, and have also been implicated as important regulators of cerebellar development^13^,^14^. Loss of N-myc increases the mRNA expression of Cyclin D2 (Ccnd2) and decreases its protein level. Ccnd2 is a key member of the cell cycle machinery that controls the transition between the G1 and S phases of the cell cycle, together with the other Cyclin D proteins (Ccnd1 and Ccnd3) and the CDKs^15^,^16^,^17^. *Ccnd2* is a well-defined c-Myc target gene^11^,^18^, and has been identified as an indirect target of Shh in cultured GCPs^19^. Loss of Ccnd2 in mice also results in decreases in cerebellar granule cell populations^13^, which is consistent with the observed phenotype in our tTS mice.

In recent years, miRNAs have been shown to play an important role in brain development. We screened miRNAs that might be involved in GCP proliferation. Our findings demonstrate that miR-9 expression is negatively responsive to N-myc expression. Loss of N-myc increases miR-9 expression, while restoration of N-myc expression could decrease miR-9 expression to normal levels. miR-9 is an ancient miRNA with a highly conserved mature sequence. In vertebrates, miR-9 is specifically expressed in the brain, and concentrates in the neurogenic regions of the embryo and adult nervous systems^20^,^21^,^22^. However, the expression of miR-9 is specifically excluded from progenitor pools of the midbrain–hindbrain boundary and rhombomere boundaries^21^,^23^,^24^, which are the regions of proliferating cerebellar GCPs^1^. This is consistent with our data showing that miR-9 expression is negatively correlated with the proliferation state of GCPs and expression of N-myc. It has been reported that *Ccnd2* is a target gene of miR-9 in mice^25^,^26^. This may explain the observed phenotype of elevated *Ccnd2* mRNA expression but reduced Ccnd2 protein level in GCPs when N-myc was inactivated postnatally. Some key transcriptional regulators controlling the progenitor state were also identified as direct targets of miR-9^24^. miR-9 inhibits Her5 and Her9 to promote neurogenesis in the developing brain in zebrafish, while miR-9 over-expression reduces the proliferation of neural progenitors^23^. However, the exact role of miR-9 in cerebellar development is unknown. Our findings indicate that over-expression of miR-9 in the cerebellum could inhibit the proliferation of GCPs by arresting the cell cycle. It has been reported that N-myc can directly bind to the promoter region of miR-9. However, under different circumstances, N-myc can regulate miR-9 in opposing ways. N-myc can up-regulate miR-9 to increase cell motility and invasiveness in breast cancer cells^27^, but it can also down-regulate miR-9 to enhance proliferation in medulloblastomas^28^. In our study, we demonstrate that miR-9 expression negatively responds to N-myc expression in GCPs. However, it remains unclear whether N-myc directly regulates miR-9 in GCPs, for example by repressing miR-9 transcription, or if this effect is indirect through post-transcriptional mechanisms or other factors.

The Shh pathway is essential for normal central nervous system development^29^, and N-myc plays essential roles as a downstream effector of this pathway in neural precursor proliferation during cerebellar development^30^. Interestingly, in the cerebellum, loss of N-myc induces a significant increase (2.7-fold) in Shh mRNA expression. *Shh* is expressed in Purkinje neurons and is secreted to promote the proliferation of GCPs^3^,^31^. Our findings suggest that the loss of N-myc in GCPs induces the arrest of their proliferation, which triggers Purkinje neurons to produce higher levels of Shh to re-stimulate this proliferation. Further exploration is needed to determine whether crosstalk and negative feedback regulation exists between Purkinje neurons and GCPs.

In summary, our results demonstrate that N-myc is a key switch regulating the proliferation cycle of postnatal cerebellar GCPs. Although further exploration of the mechanism by which N-myc regulates the proliferation of GCPs is still needed, some potential clues have emerged. N-myc can negatively regulate miR-9 expression, and miR-9 may regulate GCP proliferation by negatively regulating the expression of Ccnd2 protein. This regulation is at least partly responsible for the role of N-myc as a switch in the proliferation cycle of GCPs.

## Methods

### Animals

C57BL/6J mice and Nmyc^TRE-EGFP-Neo^ (Nmyc^EGFP^), Nmyc^TRE/TRE^, Nmyc^TRE/TRE^:tTS, Nmyc^EGFP/TRE^ and Nmyc^EGFP/TRE^:tTS mice^6^ were obtained from Shanghai Research Center for Model Organisms (Shanghai, China). The animals were housed in a specific-pathogen-free facility and maintained at 22 °C with a 12-h light/dark cycle environment. For activating and deactivating N-myc expression, mice were given 2 mg/ml DOX (Sigma-Aldrich, St Louis, MO, USA) dissolved in 5% sucrose as drinking water, which was refreshed every 2 days.

All animal experiments were approved by the Institutional Animal Care and Use Committee of Shanghai Research Center for Model Organisms and conducted in accordance with government guidelines for animal care.

### Isolation of cerebellar granule neuron precursors

Cerebellar granule neuron precursors were isolated from 7-day-old mice, as previously described^3^. In brief, cerebellum was prepared from the mice pups and washed in ice-cold phosphate-buffered saline (PBS). The meninges were removed and the cerebellum was cut into small pieces on ice and incubated in 0.25% Trypsin (Invitrogen) at 37 °C for 15–20 min. Dulbecco’s modified Eagle’s medium (10% fetal bovine serum) was added to stop Trypsin activity and tissues were triturated using pipettes to obtain a single-cell suspension. The cell suspension was passed through a 35-μm cell strainer (BD, USA) to remove debris. The cells were centrifuged at room temperature and re-suspended in ice-cold PBS. The cell suspension was underlain in a step gradient of 35% and 65% Percoll (Sigma-Aldrich) and centrifuged at high speed for 12 min. Cerebellar granule neuron precursors were harvested from the 35/65% interface. The isolated cells were washed in ice-cold PBS, centrifuged, and either TRIzol was added to extract the RNA or microRNAs, or RIPA Lysis Buffer (Beyotime, Jiangsu, China) was added to extract proteins.

### RNA extraction and qRT-PCR

Total RNA was isolated from the cerebellar granule neuron precursors by the TRIzol method (Tiangen Biotech, Beijing, China) and the cDNA was synthesised using the iScript cDNA Synthesis Kit (Bio-Rad, CA, USA). Quantitative real-time PCR was carried out using the IQ SYBR Green Supermix (Bio-Rad) and the Mastercycler Realplex2 detection system (Eppendorf, Hamburg, Germany). All primer sequences are listed in Supplementary Table S1, and the 2^−ΔΔCt^ method was used to determine relative gene expression.

### miRNA extraction and qRT-PCR

Total miRNA was isolated from the cerebellar granule neuron precursors using the miRcute miRNA kit (Tiangen Biotech), and cDNA was synthesised using the miRcute miRNA cDNA kit (Tiangen Biotech). The miRcute miRNA SYBR Green kit (Tiangen Biotech) was used for quantitative real-time PCR. The miR-9 primers were from Tiangen Biotech, and U6 was used as the internal control.

### Lentivirus microinjection

C57BL/6J mouse pups were anesthetised as previously described^32^ at 4.5 days postpartum. The lentivirus-mmu-miR-9 or control lentivirus (Genechem, Shanghai, China) were injected into the developing cerebellum by a 5-μL capacity syringe (Hamilton, Switzerland) with a 33-gauge needle. This assay required 6 μL of lentivirus stock per mouse at a titre of 10^9^ infectious units/mL. Each mouse pup had 1.5 μL of lentivirus solution microinjected per side (both the left and right cerebellum). Four days after microinjection, the pups were sacrificed for the study.

### Frozen section

Whole brains were harvested from C57BL/6J, Nmyc^EGFP^, Nmyc^EGFP/TRE^ or Nmyc^EGFP/TRE^:tTS neonatal mice and fixed in 4% paraformaldehyde overnight at 4 °C. The paraformaldehyde was then replaced by 25% sucrose, and this was renewed once after 24 h. The fixed brains were immersed in Tissue-Tek O.C.T. Compound (Sakura Finetek) for 30 min in a dark environment to replace the sucrose, and then embedded in O.C.T. at −20 °C. Ten-micrometre brain sections were cut using a cryostat (Thermo SME, USA). The sections were stained by 4′,6-diamidino-2-phenylindole, and EGFP was detected under a fluorescence microscope (Nikon 90i, Japan).

### Histological analysis

The mouse brains were harvested and fixed in 4% paraformaldehyde overnight for paraffin embedding. Sagittal sections (5 μm) were cut for HE staining. The stained sections were examined with an Eclipse 90i microscope (Nikon).

### Immunofluorescence

For analysis of the EGL proliferation and apoptosis, the frozen sections were processed according to standard immunofluorescence techniques. Polyclonal Ki-67 antibody (Abcam, USA, dilution ratio 1:200) and anti-Histone H3 (phosphor S10) antibody (Abcam, dilution ratio 1:200) were used to detect cell proliferation. Polyclonal anti-Caspase-3 antibody (Abcam, dilution 1:100) was used to detect cell apoptosis. Mouse Anti-NeuN Antibody (Millipore, USA, dilution ratio 1:100) was used to detect neuronal cells. The secondary antibodies were Alexa Fluor 555-labelled Goat Anti-Rabbit IgG (H + L), Cy3-labelled Goat Anti-Rabbit IgG (H + L) and Cy3-labelled Goat Anti-Mouse IgG (H + L) (Beyotime, Jiangsu, China), and DAPI was used to label the nuclei. The slice images were captured by intelligent laser scanning confocal microscopy (FV10i, Olympus, Japan).

### Protein extraction and western blotting

Cerebellar granule neuron precursors were added to RIPALysis Buffer (Beyotime) and put on ice for 20 min, then centrifuged at 14700 × *g* for 15 min. The supernatants were collected and the protein concentration was measured using a BCA Protein Assay Kit (Beyotime). The protein lysates were suspended in loading buffer and separated by sodium dodecyl sulfate polyacrylamide gel electrophoresis. The separated protein was transferred to a polyvinylidene fluoride membrane. The membrane was blocked and incubated with primary antibody overnight at 4 °C, then washed and incubated with secondary antibody for 1 h. The primary antibody used was Mouse monoclonal to Cyclin D2 (Abcam); the secondary antibody was IRDye 800CW Donkey anti-Mouse IgG (H + L) (Li-Cor, USA). Fluorescence was detected by Odyssey infrared imaging system (Li-Cor).

### Cell count

The whole slice images were captured by a Nikon 90i microscope. The external granular layer cells, granular layer cells, Purkinje cells and the cell area were counted using Image-Pro Plus software. At least three whole slices were counted per mouse.

### Statistical analysis

All data are expressed as mean ± SEM, and a one-way or two-way analysis of variance was used to analyse the statistical difference between groups. Significant differences were considered when *p*-values < 0.05, and in the Figures, ***p < 0.001; **p < 0.01; *p < 0.05. All the data analysis was performed using origin8.0 software.

## Additional Information

**How to cite this article**: Ma, M. *et al.* N-myc is a key switch regulating the proliferation cycle of postnatal cerebellar granule cell progenitors. *Sci. Rep.* **5**, 12740; doi: 10.1038/srep12740 (2015).

## Supplementary Material

Supplementary Information
