# Supplementary material for: N-myc is a key switch regulating the proliferation cycle of postnatal cerebellar granule cell progenitors
Source: Sci Rep. 2015 Aug 4;5:12740. doi: 10.1038/srep12740 (PMC4523855; doi:10.1038/srep12740)

N-myc is a key switch regulating the proliferation cycle of postnatal cerebellar granule cell progenitors

Ming Ma[1], Wenting Wu[2], Qing Li[1], Zhejin Sheng[1], Jiahao Shi[1], Mengjie Zhang[1],

Hua Yang[1], Zhugang Wang[2], Ruilin Sun[2]* and Jian Fei[1]*

1School of Life Science and Technology, Tongji University. Shanghai 200092, China

2Shanghai Research Center for Model Organisms, Shanghai 201203, China

Supplementary information

Supplementary table S1. The primers of Real time PCR.

Ccnd1-f	-5'-TGACTGCCGAGAAGTTGTGC -3'-
Ccnd1-r	-5'-CTCATCCGCCTCTGGCATT -3'-
Ccnd2-f	-5'-GAGTGGGAACTGGTAGTGTTG -3'-
Ccnd2-r	-5'-CGCACAGAGCGATGAAGGT -3'-
Ccnd3-f	-5'-CGAGCCTCCTACTTCCAGTG -3'-
Ccnd3-r	-5'-GGACAGGTAGCGATCCAGGT -3'-
Ccne1-f	-5'-GGTGTCCTCGCTGCTTCTGCTT -3'-
Ccne1-r	-5'-CCGGATAACCATGGCGAACGGA -3'-
CDK6-f	-5'- GGCGTACCCACAGAAACCATA-3'-
CDK6-r	-5'- AGGTAAGGGCCATCTGAAAACT-3'-

β-actin-f	-5'-CCTGTATGCCTCTGGTCGTA-3'-
β-actin-r	-5'-CCATCTCCTGCTCGAAGTCT-3'-

Supplementary Figure S1. The whole image of Western blotting assay for Ccnd2 expression in the GCPs of tTS mice and TRE mice.

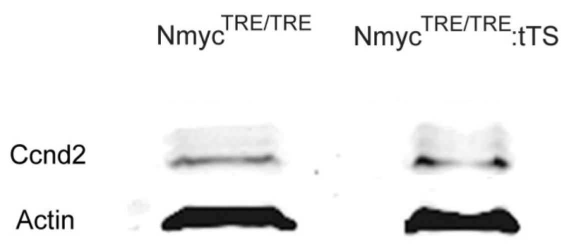

Supplement: Supplementary Information [file srep12740-s1.pdf]
